# COVID-19 Infection-Related Coagulopathy and Acute Limb Ischemia in a Patient With Pre-existing Diabetes

**DOI:** 10.7759/cureus.17531

**Published:** 2021-08-29

**Authors:** Tasnim Ahsan, Bharta Rani, Roomana S Siddiqui, Irfan Lutfi

**Affiliations:** 1 Internal Medicine: Diabetes & Endocrinology, Jinnah Postgraduate Medical Centre, Karachi, PAK; 2 Internal Medicine, Diabetes and Endocrinology, Jinnah Postgraduate Medical Centre, Medicell Institute of Diabetes Endocrinology & Metabolism (MIDEM), Karachi, PAK; 3 Family Medicine, Open Medical Institute (OMI), Karachi, PAK; 4 Interventional Radiology, Shaheed Mohtarma Benazir Bhutto Medical College, Dow University of Health Sciences, Karachi, PAK

**Keywords:** limb ischemia, coagulopathy, covid-19 infection, diabetes, anticoagulation

## Abstract

The pandemic of coronavirus 19 (COVID-19) infection has presented the clinicians with a challenge never experienced on this scale before. Although coagulopathy has been well described in association with COVID-19 infection, some recommendations have emerged so far for the potential role of empiric anticoagulation in specific situations. We describe a case of a middle-aged male with extensive acute lower limb ischemia and severe pneumonia related to COVID-19 infection. This case highlights the role of prophylactic anticoagulation in severe acute respiratory syndrome coronavirus 2 (SARS-CoV-2) infection regardless of co-morbidities and D- dimer levels.

## Introduction

The epidemic of novel COVID-19 infection caused by severe acute respiratory syndrome coronavirus 2 (SARS-CoV-2) began in Wuhan, China, in December 2019, and has spread worldwide since then [1]. The World Health Organization (WHO) declared the COVID-19 outbreak as a global pandemic on March 11, 2020 [2]. It manifests with a wide clinical spectrum ranging from asymptomatic patients to respiratory symptoms, severe pneumonia, acute respiratory distress syndrome (ARDS), septic shock and multiorgan dysfunction [3]. Approximately 20%-55% of patients with COVID-19 infection develop coagulation abnormalities, which is associated with higher morbidity and mortality [4].

## Case presentation

A 55-year-old male patient, first presented to a hospital with high-grade fever and body aches for which he was admitted. He was tested for COVID 19 RT-PCR, which was positive. The patient was shifted under our care 13 days after the onset of illness, with the complaint of worsening cough and shortness of breath over the last three days. Additionally, he had developed pain in the left calf and foot on the day of presenting to us. While in the previous hospital, he had received piperacillin-tazobactam, azithromycin, enoxaparin (60 mg subcutaneous OD) and methylprednisolone. On admission, his oxygen (O_2_) saturation was 80% on room air and increased to 92% on 10 liters of O_2_ administered through a face mask. He was fully conscious, oriented and afebrile, with a pulse of 80/min and blood pressure of 150/90 mmHg. The right foot appeared a little dusky and cold as compared to the left foot. Distal pulses in the right leg were impalpable, whereas the left leg pulses were normal. He was aware of his diagnosis of Diabetes for the last one and a half years, for which he was only taking dietary precautions and no medication. Serial laboratory tests were done as shown in Table 1.

**Table 1:** Laboratory investigations of the patient on days of illness Hb: Hemoglobin, TLC: Total leukocyte count, ALC: Absolute lymphocyte count, NLR: Neutrophil lymphocyte ratio, CRP: C-reactive protein, ESR: Erythrocyte sedimentation rate, ALT: Alanine transaminase, AST: Aspartate transaminase, ALP: Alkaline phosphatase, LDH: Lactate dehydrogenase, PCT: Procalcitonin

Parameter	Normal value	Day 1	Day 3	Day 5
Hb	13-17.5 g/dL	15.9	17.1	17.2
TLC	4-11 × 10^9^/L	12.0	17.7	25.1
ALC	1,000-4,800 cells/mm^3^	600	1,062	1,255
NLR	<3	17.8	15	18
Platelets	150-400 × 10^9^/L	162	257	322
ESR	0-13 mm/hr		20	
CRP	< 0.5 mg/dL	7.9	1.1	
Creatinine	<1.2 mg/dL	0.7	0.7	
Sodium	136-145 mEq/L	135		
Potassium	3.5-5.1 mEq/L	3.8		
ALT	<45 U/L	90	115	
rGt	<55 U/L		173	
AST	<35 U/L		73	
ALP	40-129 U/L	152	125	
PT/INR	9-14 sec/<1.1	11/0.97	11/0.95	
APTT	25-35 sec	26.5	25.5	
Ferritin	30-400 ng/mL	1,300	979.7	
D-dimer	<250 ng/mL	8,921	1,122	
LDH	120-220 U/L		625	
PCT	<0.2 ng/mL	0.23	0.06	
HbA1C	<6.5%	8.6%		
Pro-BNP	>125 pg/mL	100.7		
Albumin	3.5-5.2 g/dL	2.8		
Calcium	8.6-10.2 mg/dL	8.3		
Vit D	<30 ng/mL	10		
COVID RT-PCR		Negative		

**Figure 1:**
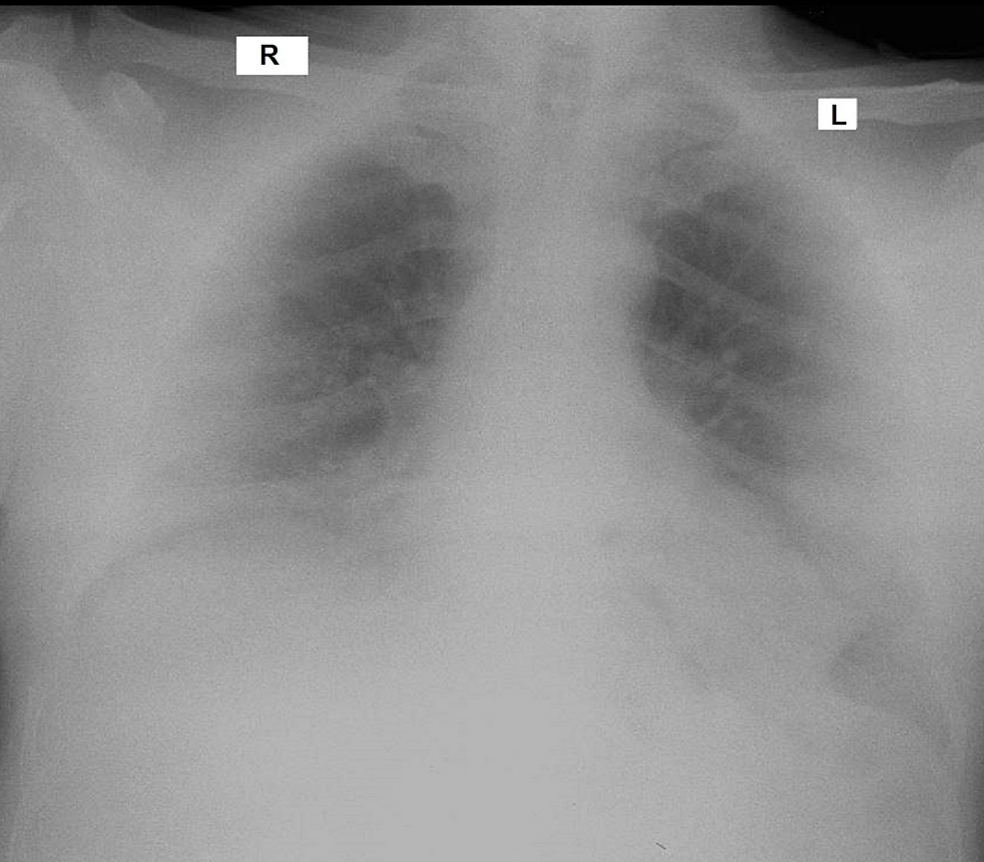
Chest x-ray showing haziness over the bases and periphery.

Chest x-ray showed a diffuse haze on the peripheral half of both lungs (Figure 1).

**Figure 2:**
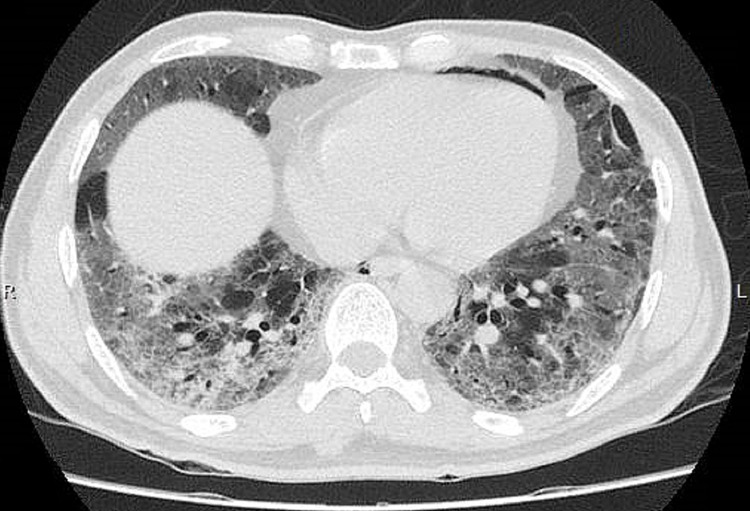
HRCT of the chest showing ground glass opacities involving both upper and lower lobes HRCT: High-resolution computed tomography

High-resolution computed tomography (HRCT) chest showed diffuse patchy airspace opacifications in both upper lobes and basal segments of lower lobes with >50% lung involvement (Figure 2).

**Figure 3:**
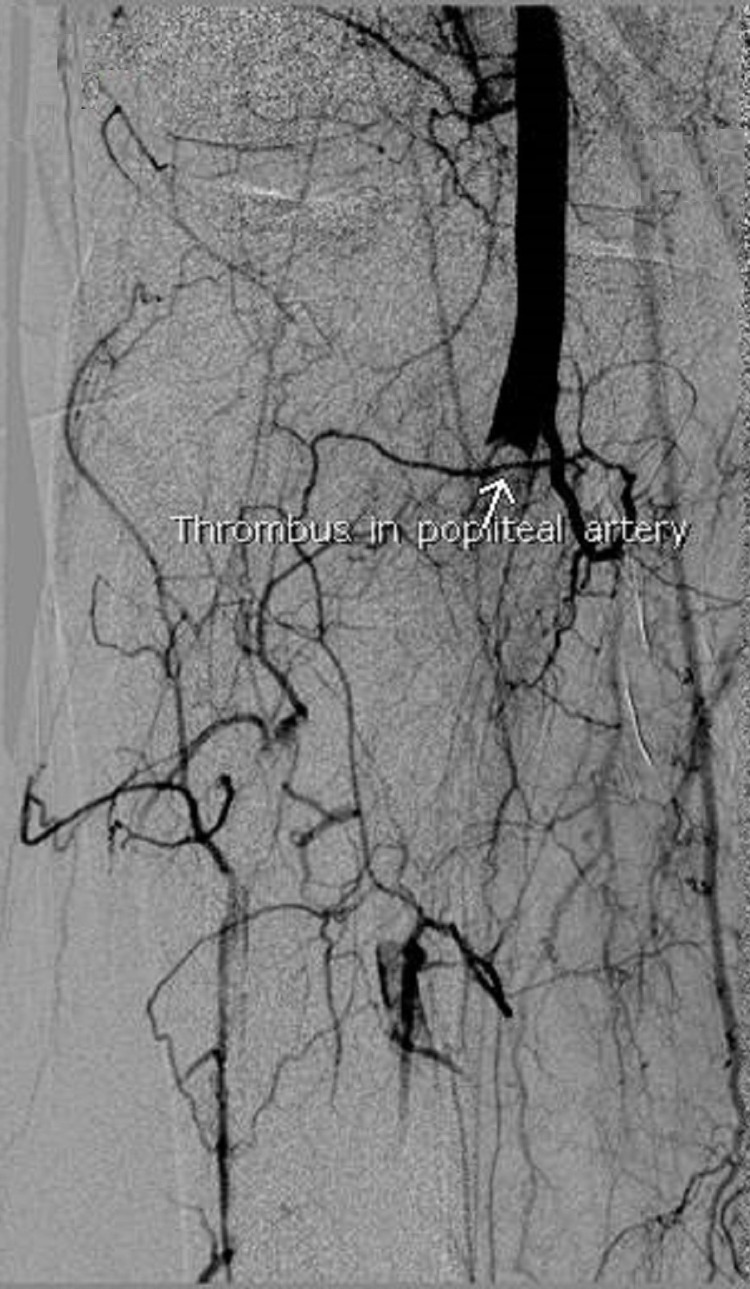
DSA showing thrombus in the right popliteal artery. DSA: Digital subtraction angiography

U/S Doppler of the right lower limb reported a triphasic pattern of flow in the femoral artery, the biphasic flow pattern in the popliteal artery and tardus parvus flow in anterior tibial, posterior tibial and dorsalis pedis arteries. Atherosclerotic changes were reported in the arteries below the knee resulting in arterial insufficiency, with a slow flow of blood to the foot with no evidence of deep vein thrombosis (DVT). Two days later, all his toes in the right foot were bluish and the tips were clearly gangrenous. Digital subtraction angiography was done at this point which showed thrombus in the P3 segment of the popliteal artery, resulting in complete occlusion and no flow was seen distal to it (Figure 3).

**Figure 4:**
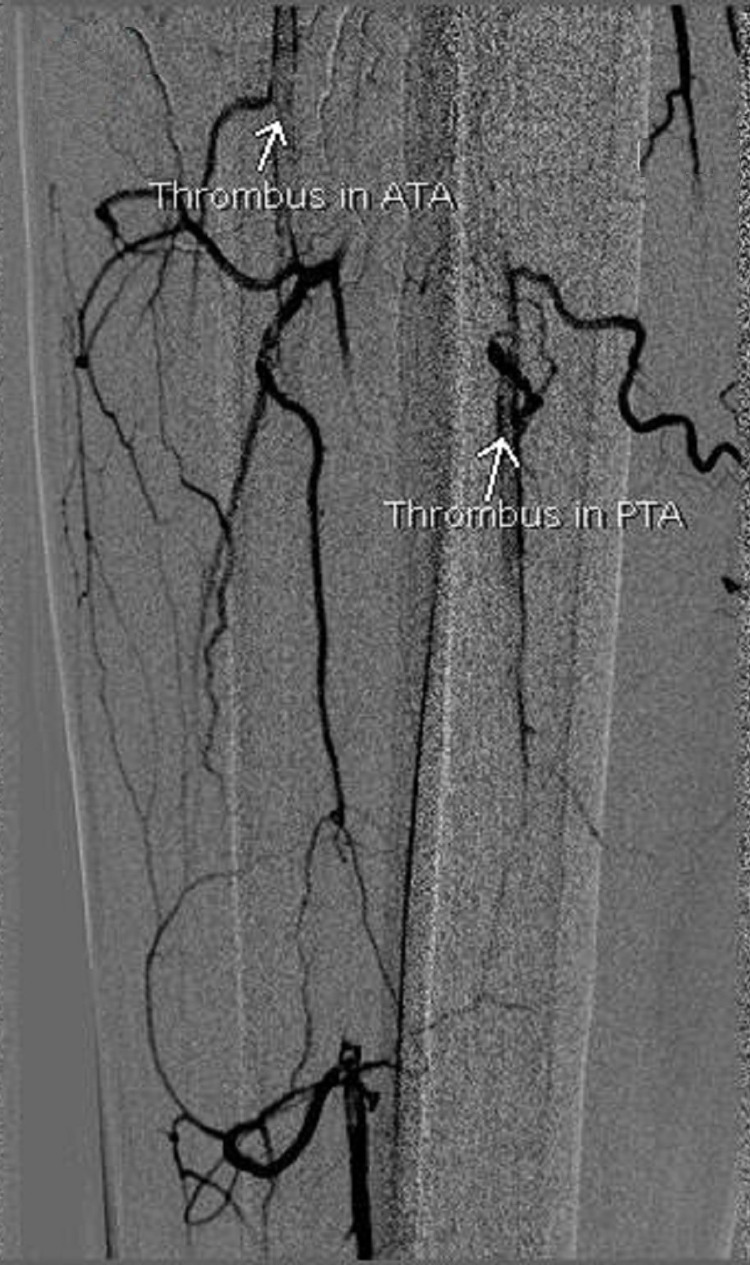
DSA showing thrombus in the right anterior tibial artery (ATA) and posterior tibial artery (PTA) DSA: Digital subtraction angiography

Thrombus was also seen in the proximal segments of anterior tibial and posterior tibial arteries, with no flow in ankle and foot, whereas weak flow was seen in collaterals in the leg region (Figure 4).

After admission, he received methylprednisolone (1 mg/kg/Qday), Enoxaparin (1 mg/kg Q12H), Insulin glargine U300 (OD) along with pre-meal regular human insulin, aspirin (75 mg OD), piperacillin-tazobactam (4.5 g Q8H), ivermectin (12 mg OD for two days), Vibramycin (100 mg Q12H for five days) cilostazol (100 mg Q12H), famotidine (40 mg Q12H), vit D3 (5,000 IU OD), vit C (500 mg Q12H), calcium gluconate (1,000 mg OD) and zinc (40 mg OD). As he was fulfilling CRS criteria, he received two doses of tocilizumab 12 hours apart (6 mg/kg/dose). The patient’s respiratory symptoms abated considerably and he was able to eat and talk without getting dyspneic; maintaining a saturation of 92% on 2-3 liters of O_2_. He was referred to a vascular surgery unit in another facility for revascularization, due to the non-availability of resources in our center, as a result of COVID-19 lockdown supply issues. Further inquiry has revealed that this patient did undergo a percutaneous angiography again, which showed extension of the thrombus in the femoral artery as well. A retrograde tibial and femoral thrombo-embolectomy was done under spinal anesthesia. Post-operatively he was reportedly doing well, but collapsed suddenly within 24 hours of the procedure and could not be resuscitated.

## Discussion

The procoagulant state has now been well recognized as a pathophysiological mechanism in the disease produced by COVID-19 infection in patients, whose disease is severe [5]. After the initial reports of raised D-dimers and fibrin degradation products, anticoagulation was recommended in the treatment protocol of all severely ill COVID-19 patients [6]. The case described here had pre-existing diabetes of short duration (18 months) and hypertension, without any symptoms attributable to lower limb ischemia prior to the current illness. His disease category was severe, largely based on his lung involvement and the need for oxygen. He received anticoagulation, steroids and tocilizumab during the hospital stay. Despite the fact that he was on a prophylactic dose of anticoagulant initially, which we had increased to a therapeutic dose on day 13, he developed extensive thrombosis in the right lower limb where the flow was already noted to be reduced. It seems that people with a pre-existing vascular disease on account of co-morbid conditions, such as diabetes and hypertension are particularly at risk of developing both arterial and venous thromboses, as reported in other cases also [7,8]. Bellosta et al. evaluated 20 patients with acute limb ischemia who were positive for COVID-19 and concluded that revascularization was lower than expected, which was hypothesized to be due to a virus-related hypercoagulable state. Therefore, it can be postulated that the use of prolonged systemic heparin may improve surgical treatment efficacy as well as limb salvage and overall mortality [9]. De novo thrombosis in people without pre-existing risk factors has also been described with SARS-CoV-2 infection. Perini et al. reported two young non-atherosclerotic patients with COVID-19 infection, presenting with acute limb ischemia; one had recurring thrombosis and could not survive despite thrombo-embolectomy; the other responded well to unfractionated heparin [10]. Spontaneous COVID-19 infection-related arterial coagulopathies have been described with grossly elevated D-dimers as well as with mild elevation [11]. Therefore, it can be concluded that it might be beneficial to consider prophylactic anticoagulation for an extended period of time (two to four weeks) in all patients hospitalized with moderate/severe and critical COVID-19 infection, rather than an absolute D-dimer level cut-off level to initiate anticoagulation.

## Conclusions

Based on an increasing number of case reports documenting the occurrence of coagulopathy in COVID-19 infection, it may be deduced that prophylactic anticoagulation should be instituted in all patients, rather than only those who show a D-dimer elevation of ≥ 3 times the upper limit normal or who have pre-existing vascular disease.
